# The social gradient in adolescent unintentional injuries demands policy attention

**DOI:** 10.1186/s40621-026-00728-8

**Published:** 2026-11-13

**Authors:** Sofie Kruckow, Amy E. Peden, Ulrik Becker, Janne S. Tolstrup

**Affiliations:** 1 National Institute of Public Health, University of Southern Denmark, Studiestraede 6, Copenhagen K, DK-1455 Denmark; 2 School of Population Health, Faculty of Medicine and Health, UNSW Sydney, Kensington, NSW 2052 Australia; 3 Discipline of Public Health and Tropical Medicine, College of Public Health, Medical and Veterinary Sciences, James Cook University, Townsville, Queensland Australia

**Keywords:** Adolescent health, Unintentional injury, Social determinants of health, Public health policy, Injury prevention

## Abstract

Unintentional injuries are a leading cause of morbidity and mortality in adolescence, yet they remain underprioritised within adolescent health and equity agendas. Recent Danish studies reveal substantial socioeconomic inequalities in unintentional injuries. Injury risk and injury-related mortality follow a clear social gradient, with adolescents from less advantaged backgrounds experiencing higher rates of both outcomes. Despite these disparities, evidence on effective and equitable prevention strategies remains limited, and current policy efforts largely focus on road traffic injuries while overlooking other important causes. Furthermore, existing injury surveillance systems inadequately capture adolescent-specific contexts and activities, limiting opportunities for targeted prevention. Unintentional injuries are an important but overlooked source of health inequality in adolescence. Reducing the burden of injury requires greater policy attention, stronger research and surveillance, and a focus on the social determinants that drive injury risk. Addressing these upstream factors may improve both injury outcomes and adolescent health more broadly.

Adolescents comprise 25% of the global population and play a pivotal role in shaping future societies and economies; however, investment in adolescent health remains disproportionately low [1]. In October 2025, the WHO European Region introduced a new strategy to promote child and adolescent health and well-being (*A Healthy Start for a Healthy Life: A Strategy for Child and Adolescent Health and Well-being in the WHO European Region 2026–2030*) [2, 3]. Although the strategy identifies health equity as an urgent priority for reducing preventable disease and social exclusion, it fails to address unintentional injuries with an equity lens. This omission highlights the lack of attention to social determinants in injury prevention and underscores their untapped potential for promoting adolescent health, particularly given that unintentional injuries are one of the leading causes of morbidity and mortality in adolescents [4].

While socioeconomic inequalities in injury morbidity and mortality are relatively well established internationally, much of the evidence is derived from pediatric populations [5]. Recent Danish studies have extended this evidence base by providing comprehensive and contemporary data on adolescents aged 15–24 years. In the Danish National Youth Cohort, which followed 70,342 adolescents aged 15–24 years from 2014 to 2022, 40% sustained at least one unintentional injury requiring hospital care, with 4% classified as severe injuries [6]. Injury rates were 14–34% higher among the least socio-economically advantaged compared to the most advantaged adolescents. Moreover, a clear dose–response relationship was observed across the full spectrum of parental education, income, and occupational status. Notably, 11% of adolescents accounted for 50% of all injuries, and those at the lower end of the social gradient were much more likely to belong to this high-risk subgroup.

The socioeconomic pattern in injury risk also manifests in the mortality related to unintentional injuries. In a cohort study using Danish nationwide data, we analysed all deaths from 2010 to 2022 among 15–24-year-olds and found a clear socioeconomic gradient in unintentional injury deaths, with more than a three-fold higher mortality rate in the least advantaged compared to the most advantaged adolescents [7]. This social gradient exposes the heavy toll of unintentional injury, particularly among those already burdened by socioeconomic disadvantage.

The clear social gradient in injury risk and injury-related mortality demonstrates systematic inequalities, suggesting that structural prevention efforts could be effective. However, aside from interventions targeting sports injuries, knowledge on effective measures remains sparse and is largely limited to high-income countries, with low equity considerations [8]. Thus, while the current evidence base suggests that structural prevention may hold promise as an effective approach to reducing injury disparities in adolescence, robust evidence for such initiatives, particularly in the populations most in need, is still sorely lacking.

At the policy level, traffic-related injuries tend to dominate attention, evidenced by the fact that only road traffic accidents are explicitly addressed by the United Nations Sustainment Development Goals (Target 3.6), rendering other types of unintentional injuries, such as drowning, falls and poisoning among others, largely invisible [9]. Traffic injuries account for a substantial proportion of unintentional adolescent injury deaths (58%) and disability-adjusted life years (52%) [4]. However, unintentional injuries encompass a broad spectrum of non-traffic incidents, including those arising from leisure activity, sports, work, play, and domestic duties among others. Collectively, they accounted for 10% of all deaths among 10-24-year-olds globally in 2019, most often due to drownings and falls [4].

Established by the Nordic Medico-Statistical Committee (NOMESCO) [10] the above mentioned categories represent child and adult activities yet they fail to capture the strong orientation toward peer-related activities that are common in adolescence. NOMESCO offer a standardised system applied throughout the Nordic region (Denmark, Finland, Iceland, Norway, and Sweden) to document and analyse external causes of injuries and accidents. It is mandatory to register the activity leading to injury for all hospital contacts due to an accident. However, activities such as spending time with friends, attending parties, and other peer-influenced risk-taking situations are not represented and may be grouped under broad categories such as “leisure activity” or “entertainment,” or simply registered as “other” limiting insight into adolescent-specific injury mechanisms. In 30% of unintentional injury cases in The Danish National Youth Cohort, the activity was undetermined, leaving a significant gap in understanding the circumstances behind these injuries [6].

Social determinants (e.g., parental income, education, and occupation) shape the conditions in which adolescents live and grow thereby influencing a wide range of health and well-being outcomes. Addressing the social determinants of unintentional injuries therefore represents an unexploited lever with potential dual wins for adolescent health and well-being by delivering gains beyond injury prevention. Investing in adolescent health benefits young people today, the adults they will become, and future generations. This positions the social determinants as a strategic driver that we can neither afford to forget nor underprioritize.

To advance adolescent injury prevention we call for action recognizing the following three points: 1) prioritise injury prevention 2) advance injury research and 3) adress social determiants of health.

## Prioritise injury prevention

To ensure that adolescents have equitable opportunities for a long and healthy life, injury prevention must be recognised as a central and indispensable priority for policymakers, professionals, researchers, and advocates for adolescent health with the aim of moving from awareness to action. Greater traction may be achieved by embedding injury prevention within broader adolescent health agendas, recognizing injuries as a health equity issue given their pronounced social gradient, strengthening surveillance systems to better capture the social and contextual determinants of adolescent injury, and generating stronger economic evidence on healthcare costs, productivity losses, and long-term consequences to support policy prioritization.

## Advance injury research

Although socioeconomic inequality has long been recognised in injury prevention, empirical evidence on whether prevention strategies effectively reduce these inequalities remains scarce, despite strong evidence that such interventions reduce injury rates overall, even for road traffic injuries, which is a relatively mature area of injury prevention. The limited equity-focused evidence in this comparably well-developed field highlights the even greater evidence gap regarding equity impacts for other injury mechanisms. Future research should evaluate the effects of injury prevention interventions across socioeconomic groups to determine whether they reduce, maintain, or widen existing inequalities. Furthermore, research should pay attention to the pathways linking individual (substance use, mental health, experimental behavior) and contextual (peer influence, family environment, neighborhood deprivation) risk factors to the broader social determinants that generate injury inequalities. This could be achieved through multilevel analyses combining individual and contextual data, as well as mixed-methods studies. To support this work, injury surveillance systems must capture adolescent-specific activities and contexts, including social settings, peer involvement, and circumstances preceding injury while allowing for suitable disaggregation by age group.

## ​Adress social determinants of health

Adolescent injuries are prevalent and follow a pronounced social gradient, ultimately resulting in excess mortality from injuries. Social determinants, also referred to as the “causes of the causes,” are upstream, structural drivers of more proximal causal risk factors and thus represent an important approach to injury prevention. Social reforms that create more equitable societies may reduce the underlying social gradient in injury risk, including free and equitable education, redistributive policies, affordable housing, and investments in safe neighborhood environments. However, empirical evidence demonstrating the effectiveness of such policies in reducing adolescent injury inequalities remains limited.

**Fig. 1:**
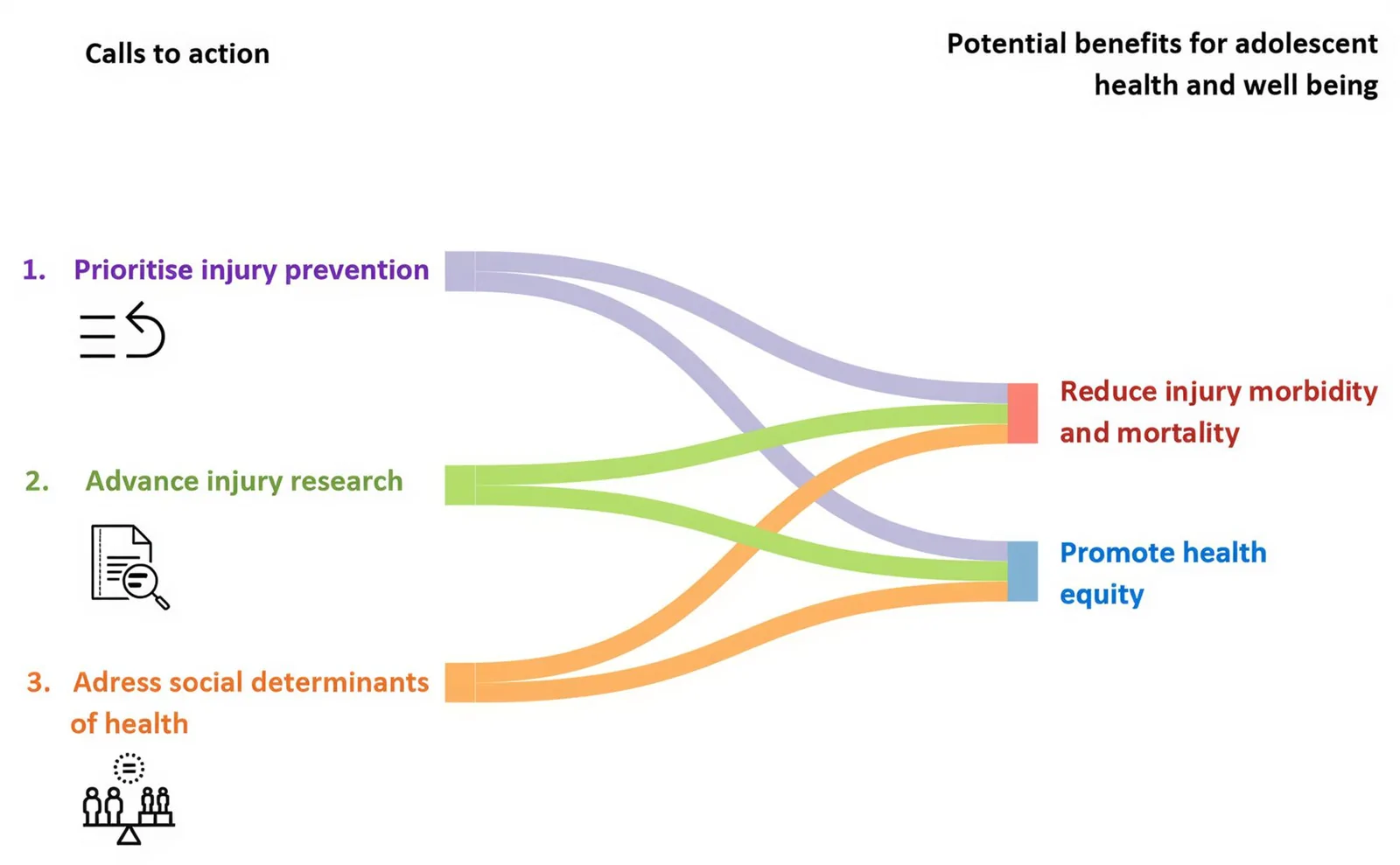
Calls to action. Pronounced negative social gradients in adolescent injury highlight two interconnected opportunities for health promotion. On the one hand, prioritising injury prevention and research at the political level can directly reduce substantial morbidity and mortality while advanc­ing health equity. On the other hand, addressing the social determinants targets the root causes of injury inequalities, with the potential to promote health equity more broadly

Beyond reducing injuries, acting on these priorities represents an opportunity to promote health equity more broadly, as illustrated in Fig. 1. Adolescence is a critical window for injury prevention given the substantial burden that injuries place on health and well-being during this period. Failing to address these inequalities risks missing an important opportunity to improve lifelong health and reduce inequities across the life course and generations.

## Data Availability

No datasets were generated or analysed during the current study.
